# An accelerated miRNA-based screen implicates Atf-3 in *Drosophila* odorant receptor expression

**DOI:** 10.1038/srep20109

**Published:** 2016-02-05

**Authors:** Shreelatha Bhat, Walton D. Jones

**Affiliations:** 1 Lab for Behavioral Neurobiology, KAIST Biological Sciences, 291 Daehak-ro, Yuseong-gu, Daejeon, 34141, Republic of Korea.

## Abstract

The *Drosophila* olfactory system is highly stereotyped in form and function; olfactory sensory neurons (OSNs) expressing a specific odorant receptor (OR) always appear in the same antennal location and the axons of OSNs expressing the same OR converge on the same antennal lobe glomeruli. Although some transcription factors have been implicated in a combinatorial code specifying OR expression and OSN identity, it is clear other players remain unidentified. In hopes of mitigating the challenges of genome-wide screening, we examined the feasibility of a two-tiered approach comprising a primary “pooling” screen for miRNAs whose tissue-specific over-expression causes a phenotype of interest followed by a focused secondary screen using gene-specific RNAi. Since miRNAs down-regulate their targets, miRNA over-expression phenotypes should be attributable to target loss-of-function. It is the sequence-dependence of miRNA-target pairing that suggests candidates for the secondary screen. Since miRNAs are short, however, miRNA misexpression will likely uncover non-biological miRNA-target relationships. Rather than focusing on miRNA function itself where these non-biological relationships could be misleading, we propose using miRNAs as tools to focus a more traditional RNAi-based screen. Here we describe such a screen that uncovers a role for Atf3 in the expression of the odorant receptor Or47b.

The transgenic RNAi fly stock libraries (e.g., the Vienna Drosophila RNAi library^1^ and the Transgenic RNAi Project (TRiP)) have been a tremendous boon to the *Drosophila* community because they permit tissue-specific knockdown of almost all genes in the genome. These resources permit genome-wide screens for genes associated with almost any phenotype of interest. Unfortunately, the sheer size of these libraries—more than 22,000 stocks in the case of the Vienna library—means performing such screens remains labor-intensive and tedious. In this paper, we describe our development of a two-tiered screening protocol comprising an initial pooling screen using miRNA over-expression that generates a list of candidate genes involved in a phenotype of interest and a secondary screen using gene-specific RNAi that narrows this list of candidates to the responsible target gene(s). We suggest that this protocol can sometimes accelerate the identification of novel genes involved in a broad range of phenotypes.

MicroRNAs are short, endogenous, single-stranded RNA molecules that act in the context of the miRISC protein complex to either inhibit translation or induce the degradation of target mRNAs^2^. Since the miRNA-target mRNA relationship is determined primarily by a short seed sequence at the 5′ end of each miRNA^3^,^4^, the complement of which may occur in multiple copies scattered over the genome, many miRNAs are capable of down-regulating multiple targets. The relationship between a miRNA seed sequence and its complements in the open reading frames and 3′-untranslated regions (3′-UTRs) of target mRNAs spurred the development of bioinformatic algorithms that convert mature miRNA sequences into lists of potential mRNA targets^5^. These lists of candidate targets, however, are plagued by large numbers of false positives because the algorithms that generate them can fully account for neither the precise spatial and temporal patterns of miRNA and target mRNA expression nor target site availability. In other words, a miRNA may be capable of down-regulating a particular target and never actually do so, either because the two are never simultaneously expressed in the same tissue or because RNA-binding proteins or RNA folding render the target site inaccessible. It also follows that miRNA over-expression in arbitrary tissues using the binary GAL4/UAS expression system would likely lead to non-biological miRNA-target mRNA pairings. Rather than seeing these pairings as a potential drawback of using a library of UAS-miRNA stocks, we expect they can be useful as part of a two-tiered screening system.

We previously generated a library of 131 UAS-miRNA fly stocks that permit tissue-specific over-expression of 144 *Drosophila* miRNAs^6^. In this study, we sought to use these UAS-miRNA stocks to validate the concept of a two-tiered miRNA-based screen in the *Drosophila* olfactory system.

The olfactory sensory neurons (OSNs) of adult *Drosophila* are housed in porous hair-like sensilla that cover the paired antennae and maxillary palps. Olfactory sensilla are divided into 3 main classes by their shape and 17 subclasses by their odor response profiles^7^. The odor response profile of an OSN is determined by its expression of the obligatory olfactory co-receptor Orco and one or very few of the adult odor-specific odorant receptors (ORs)^8^. The spatial arrangement of the 17 subclasses of adult olfactory sensilla on the antenna, the arrangement of the OSNs themselves, the precise pattern of OR expression, and the wiring of the antennal OSNs into the appropriate glomeruli of the antennal lobe are all highly stereotyped from fly to fly, indicating well-orchestrated developmental control of every step in the process.

Jafari *et al.* reported the results of a large-scale RNAi screen that identified seven transcription factors, permutations of which determine the odorant receptor expressed by each population of olfactory neurons in the adult *Drosophila* antenna. Despite the success of their screen, Jafari *et al.* extrapolated from the complexity of the fly olfactory system and estimated that at least three more unidentified transcription factors are likely part of the combinatorial code that determines OR expression^9^.

In their screen, Jafari *et al.* combined the Peb-GAL4 driver line, which is strongly expressed in peripheral sensory neurons including the antennal olfactory neurons, with a pair of OR promoter fusions (i.e., Or47b and Or92a) to a membrane-tethered GFP that act as reporters of OR expression. We obtained these lines and by crossing them to our library of UAS-miRNA stocks we were able to identify miRNAs whose over-expression eliminates Or47b expression, Or92a expression, or both. We chose to proceed with the miRNAs that affect Or47b expression (i.e., bantam, miR-2a-2, miR-33, miR-263a, miR-308, miR-973/974, and miR-2491). We then used existing bioinformatic tools to generate lists of their putative mRNA targets, compare the lists for overlap, and define a short list of candidate genes for a small follow-up RNAi screen. In this follow-up screen, we identified a previously unknown role for Activating transcription factor 3 (Atf3) in the expression of Or47b.

## Results

### Two-tiered miRNA-based screening

**Figure 1:**
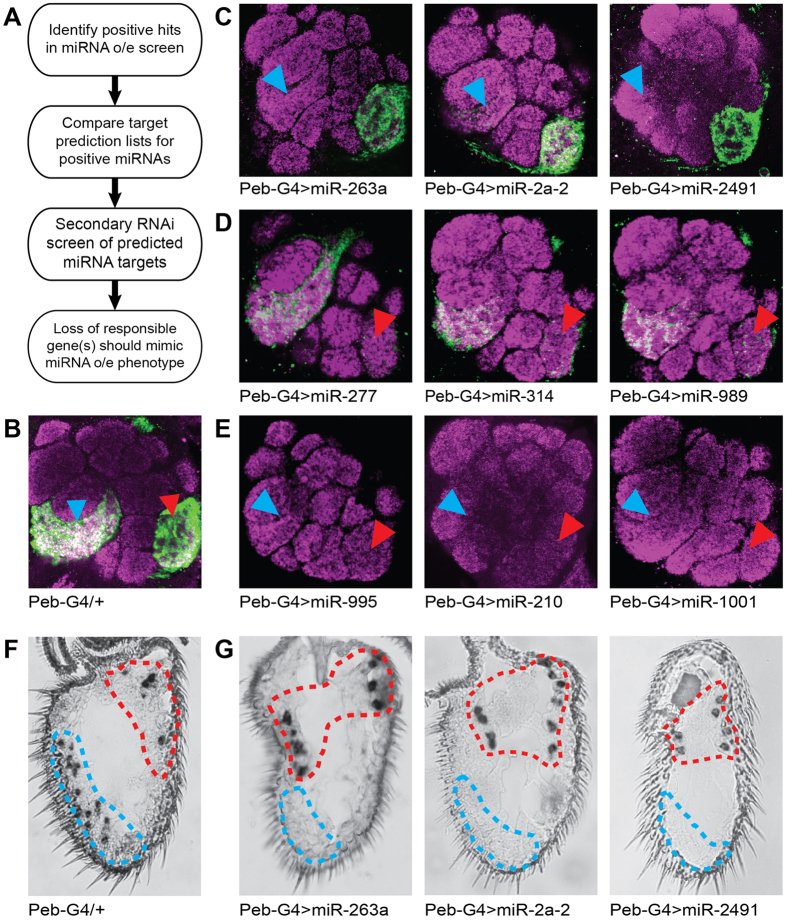
Primary screen for miRNAs whose over-expression alters OR reporter expression in the antennal lobe. (**A**) A two-tiered, miRNA-based screening workflow can accelerate loss-of-function genetic screens by limiting the number of necessary gene-specific RNAi crosses. (**B**) Antennal lobe (AL) staining of the Peb-GAL4/+; Or47b>CD8::GFP, Or92a>CD8::GFP/+ control shows strong GFP staining in both the Or47b glomerulus VA1lm (blue arrowhead) and the Or92a glomerulus VA2 (red arrowhead). (**C**) AL staining for 3 of the 7 miRNAs whose over-expression induces loss of the Or47b reporter (blue arrowheads). (**D**) AL staining for 3 of the 4 miRNAs whose over-expression induces loss of the Or92a reporter (red arrowheads). (**E**) AL staining for the 3 miRNAs whose over-expression induces loss of both the Or47b and Or92a reporters (blue and red arrowheads). (**F**) Mixed probe *in situ* on the same control genotype as in B. Or92a-expressing cells are circled in red, while Or47b-expressing cells are circled in blue. (**G**) Mixed Or47b/Or92a probe *in situs* on antennae of the genotypes shown in D confirm the loss of Or47b expression induced by miR-263a, miR-2a-2, and miR-2491.

Three other collections of UAS-miRNA stocks published recently were presented as tools either for identifying novel miRNA functions^10^,^11^ or for use in the context of screens for modifiers of existing developmental phenotypes^12^. Rather than using the UAS-miRNA lines we generated^13^ to study miRNA biology, we asked whether they could also be used as a tool permitting a “pooling” pre-screen designed to limit the focus and thus accelerate a secondary, but more traditional RNAi-based loss-of-function screen. In such a scheme, after identifying miRNAs whose over-expression induces a phenotype of interest, bioinformatic target prediction provides a list of candidate genes for a follow-up RNAi screen. Since miRNAs inhibit the translation of or induce degradation of their target mRNAs, phenotypes observed upon miRNA over-expression should be replicable via RNAi knockdown of the responsible target(s). Figure 1A presents a generalized flowchart of this two-tiered screening strategy.

### Primary screen for miRNAs that modify OR expression

As stated, we settled on *Drosophila* olfactory neurons to demonstrate our miRNA-based screening strategy. We chose Peb-GAL4 to drive expression of UAS-miRNAs in adult olfactory neurons because it is expressed strongly in peripheral sensory neurons beginning 12–18 hours after pupation^14^. By combining Peb-GAL4 with fusions of two odorant receptor promoters (i.e., Or92a and Or47b) to the coding sequence for a membrane-tethered GFP and crossing in the UAS-miRNAs, we were able to visualize the effects of miRNAs on OR expression. Or92a is expressed in large basiconic sensilla in a class of OSN called ab1B, which respond to 2,3-butanedione^15^. Or47b is expressed in trichoid sensilla in a class of OSNs called at4, which respond to socially relevant fly-derived odors^16^. We chose Or92a and Or47b because they are expressed in olfactory neurons that innervate prominent, well-separated glomeruli in the antennal lobe (i.e., VA2 and VA1lm) and whose cell bodies are easily distinguishable upon antennal RNA *in situ* hybridization.

For the primary miRNA over-expression screen, we crossed a stable homozygous stock containing Peb-GAL4 and the Or47b and Or92a promoter fusions to all of our UAS-miRNA lines. We observed that Peb-GAL4-mediated over-expression of 20 of 131 miRNA lines is lethal or nearly lethal (see Supplemental Table 1). For the remaining miRNAs, we compared the levels of the GFP reporters of OR expression in the antennal lobes of flies over-expressing miRNAs with a heterozygous control (Fig. 1B). In doing so, we were able to divide miRNAs into three categories: those that reduce the intensity of the Or47b reporter in VA1lm (Fig. 1C, blue arrowheads), the Or92a reporter in VA2 (Fig. 1D, red arrowheads), or both (Fig. 1E).

We decided to focus our follow-up on the miRNAs that reduce the expression of the Or47b reporter because only one transcription factor, Eip93f, is associated with Or47b expression^9^. In addition, some of the miRNAs that cause loss of Or92a also cause a less significant loss of Or47b, meaning the loss of Or47b phenotypes are less ambiguous. Before proceeding to miRNA target identification, we verified the miRNA-induced loss of Or47b using RNA *in situ* hybridization with mixed riboprobes against both Or47b and Or92a. Combined we identified seven miRNA lines (Pictured: miR-263a, miR-2a-2, and miR-2491; Not shown: bantam, miR-33, miR-308, and miR-973/974) that reduce the expression of the Or47b reporter. We were able to verify that these lines eliminate Or47b (circled in blue) expression when combined with the Peb-GAL4 driver while leaving Or92a (circled in red) expression intact (compare Fig. 1F,G).

### Secondary RNAi screen for miRNA targets that modify OR expression

**Figure 2:**
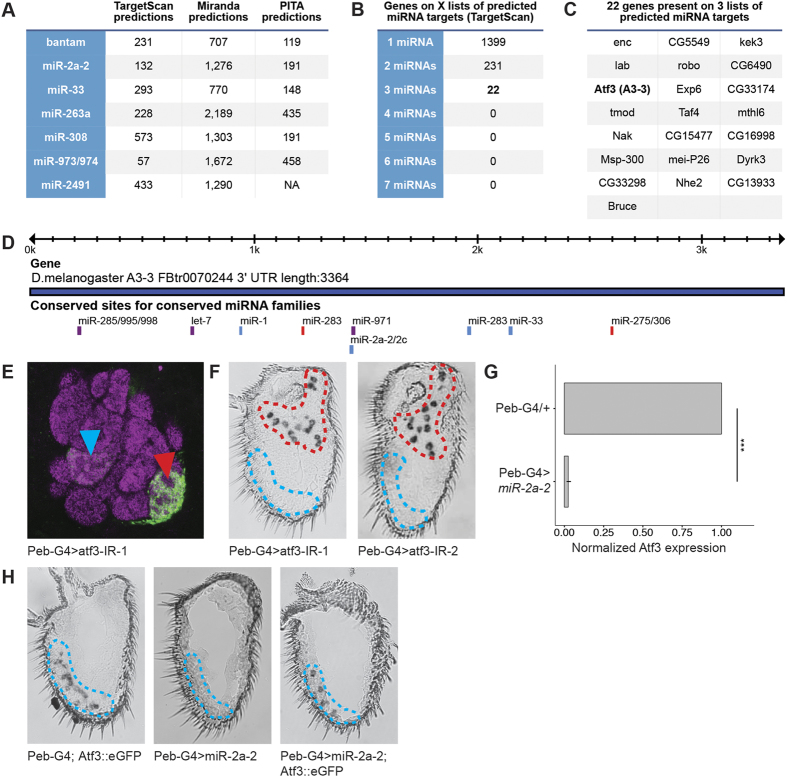
Secondary screen for regulators of Or47b expression using gene-specific RNAi. (**A**) Tabular summary of the target predictions made by three different algorithms for each miRNA whose over-expression reduces Or47b expression. (**B**) The number of genes that appear on the TargetScan candidate target lists in A. (**C**) Twenty-two genes, including Atf3, appear on the candidate target lists for 3 different miRNAs. (**D**) A schematized view of the Atf3 (A3-3) 3′-UTR showing the locations of seed matches for miR-2a and miR-33, which reduce Or47b expression. (**E**) Atf3 knockdown in all olfactory neurons using Peb-GAL4 reduces expression of an Or47b reporter, but spares expression of an Or92a reporter. (**F**) Antennal *in situ* hybridization with mixed riboprobes that recognize Or47b (blue) and Or92a (red) reveals that Atf3 knockdown using two different RNAi lines eliminates Or47b expression, but spares Or92a expression. (**G**) Over-expression of miR-2a-2 in olfactory neurons using Peb-GAL4 reduces the expression of Atf3 in the antenna. n = 2 biological replicates of 3 technical replicates each. ***p = 0.0004. (**H**) An Atf3::eGFP fusion protein lacking the Atf3 3′-UTR, which contains the miR-2a-2 binding site, rescues the loss of Or47b expression observed with miR-2a-2 over-expression.

Following the screening protocol outlined in Fig. 1A, we next compiled lists of possible target genes for each of the miRNAs whose over-expression eliminates Or47b expression. We initially compared the outputs of multiple target prediction algorithms (i.e., TargetScan, miRanda, and PITA), as this is standard practice^17^,^18^. Figure 2A compares the numbers of candidate targets predicted for each miRNA that induces loss of Or47b by the TargetScan^19^, miRanda^20^, and PITA^21^ algorithms.

Typically, miRNA-based screens using GAL4 lines that drive expression in well-characterized tissues would allow a list of predicted miRNA targets to be prioritized based on expression in the tissue of interest. In this case, however, without access to transcriptome data for the olfactory neurons of interest, we decided to prioritize our secondary RNAi screen by looking for overlap between the lists of candidate targets for each miRNA whose over-expression eliminated Or47b expression. We decided to focus on TargetScan because it predicts miRNA seed matches in both 3′-UTRs and open reading frames as well as provides a way to query more recently identified miRNAs like miR-2491. PITA does not provide easy access to this option, and the miRanda algorithm produces too many predicted targets. TargetScan predicts a total of 1399 unique targets for the seven UAS-miRNA lines that eliminate Or47b expression. While 231 of these unique candidates are shared between the prediction lists for two of the miRNAs, only 22 targets appear on the lists for three of the miRNAs (Fig. 2B,C).

We next screened through this list of candidate targets appearing on the prediction lists for multiple miRNAs by combining gene-specific UAS-candidate-IR (inverted repeat) lines with the control Peb-GAL4; Or47b>CD8::GFP, Or92a>CD8::GFP genotype. In this secondary screen, knockdown of responsible targets can be expected to mimic the miRNA-induced loss of Or47b. This is how we were able to identify a role for Activating Transcription Factor 3 (Atf3 or A3-3) in the expression of Or47b. Atf3 appears on the predicted target lists for three miRNAs whose over-expression eliminates Or47b expression (i.e., miR-2a-2, miR-33, and miR-2491). Figure 2D indicates the positions of the miR-2a-2 and miR-33 binding sites in the Atf3 3′-UTR. The miR-2491 binding site in the 3′-UTR of Atf3 is conserved across several species of *Drosophila*, but because it is not part of the standard TargetScan search parameters it does not appear in the exportable graphic used to make Fig. 2D. Note also that over-expression of several miRNAs (e.g., let-7, miR-285, etc.) with predicted binding sites in the Atf3 3′-UTR fails to eliminate Or47b expression, presumably due to issues with binding site accessibility. This fact that it is currently impossible to predict which of the clear miRNA seed match sites represent true positives is what makes a follow-up gene-specific RNAi screen necessary. Figure 2E shows the result of our secondary RNAi screen in which knockdown of Atf3 dramatically and specifically reduces the expression of the Or47b reporter (blue arrowhead) and not the Or92a reporter (red arrowhead). Furthermore, loss of Atf3 eliminates Or47b expression as assessed via mixed probe *in situ* hybridization while leaving Or92a expression unaffected (compare Figs 1G and 2F). Since we are using miRNAs as a screening tool, the precise mechanisms by which they produce their phenotypes are irrelevant to the results of the follow-up screen. Still, in the interest of completeness, we confirmed that the over-expression of miR-2a-2 reduces the level of Atf3 in antennal cDNAs as detected by qPCR (Fig. 2G). We were also able to confirm that the loss of Or47b expression induced by miR-2a-2 over-expression is rescued by introduction of an Atf3::eGFP fusion that lacks its 3′-UTR and thus the miR-2a-2 binding site (Fig. 2H).

It is interesting to note that Eip93f, the only other transcription factor whose loss-of-function eliminates Or47b expression, appears on the candidate target lists of two miRNAs whose over-expression eliminates expression of the Or47b reporter (i.e., miR-308 and miR-2491). This means we could have identified it in a second round of gene-specific RNAi screening had the list of candidate targets appearing on three lists failed to identify a true positive.

### Validation of a role for Atf3 in Or47b expression

**Figure 3:**
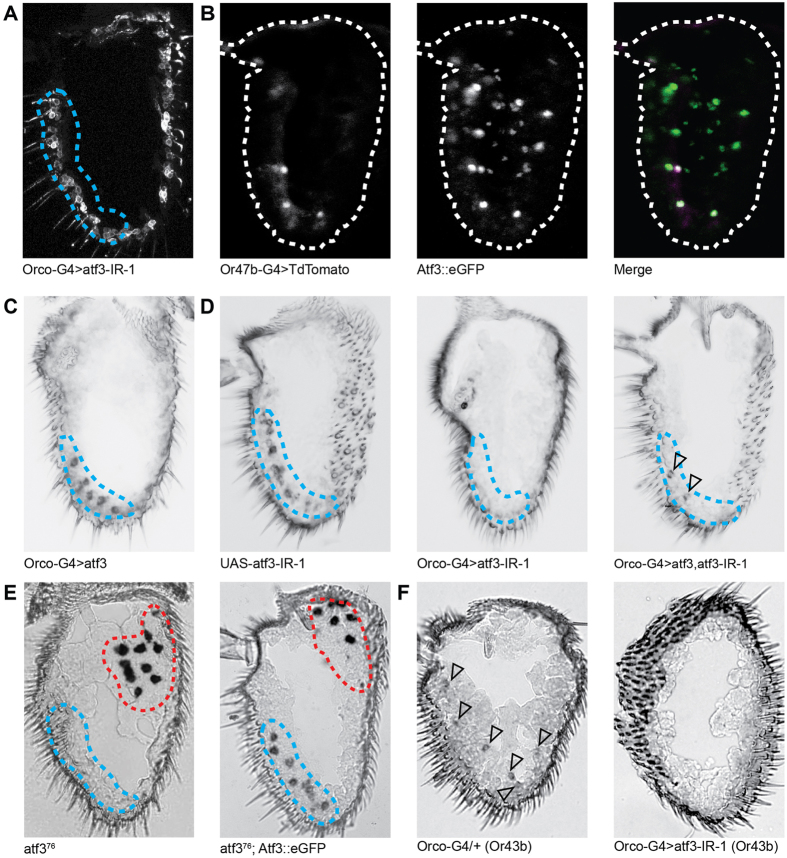
Atf3 is required for the expression of Or47b. (**A**) Orco-GAL4 driven knockdown of Atf3 does not affect the morphology of the Or47b neurons, nor does it affect Orco expression or dendritic localization. (**B**) An Atf3::eGFP protein fusion under the control of the Atf3 promoter is co-expressed with Or47b-GAL4 driving UAS-TdTomato in Or47b neurons. (**C**) Over-expression of Atf3 in most adult OSNs using Orco-GAL4 does not expand the expression of Or47b. (**D**) When compared to the UAS-atf3-IR-1 control (left), Orco-GAL4 driven knockdown of Atf3 eliminates Or47b expression (center), but this can be partially rescued by UAS-Atf3 (right). Rescued cells are indicated with open triangles. (**E**) (Left) Atf3-null atf3^76^ mutant antennae show normal Or92a expression (circled in red), but lack Or47b expression (circled in blue). (Right) The Atf3::eGFP transgene rescues Or47b expression in atf3^76^ antennae. (**F**) Knock-down of Atf3 in most OSNs using Orco-GAL4 eliminates Or43b expression. Or43b-positive cells are indicated with open triangles.

Atf3 is a member of the basic leucine zipper (bZIP) family of transcription factors. In *Drosophila*, Atf3 functions in immune and metabolic homeostasis as well as abdominal morphogenesis^22^,^23^. Consistent with a role for Atf3 in Or47b expression rather than olfactory neurogenesis, knockdown of Atf3 does not affect OSN morphology, Orco expression, or Orco localization (Fig. 3A). We also confirmed that Atf3 is expressed in Or47b neurons by combining an Atf3 protein fusion to eGFP under the transcriptional control of the Atf3 promoter (Atf3::eGFP) with an Or47b-GAL4 line driving the red fluorescent marker UAS-TdTomato (Fig. 3B).

The fact that the other transcription factors implicated in OR expression (e.g., Acj6, Pdm3, Xbp1, Eip93f, etc.) work together as part of a combinatorial code that includes both transcriptional activators and repressors^9^, suggests that broad antennal over-expression of Atf3 may expand the expression pattern of Or47b. On the contrary, the combination of UAS-Atf3 with the olfactory co-receptor Orco-GAL4 line, which drives expression in most antennal OSNs, has no effect on the Or47b expression pattern (Fig. 3C). This suggests other OSN populations express sufficient repressive transcription factors to prevent Or47b expression or lack an Atf3 binding partner required for Or47b activation. It is also possible that chromatin modifications or other unknown changes arise between OSN birth and final differentiation that somehow prevent the functional binding of ectopic Atf3 to the promoters of other ORs.

We used Peb-GAL4 in our two-tiered miRNA-based screen because it drives expression in peripheral sensory neurons including those of the developing antenna beginning 12–18 hours after puparium formation (APF)^14^. Since Peb-GAL4 expression begins soon after the birth of the OSNs and long before the earliest OR expression begins at 50 hours APF, it is possible that Atf3 plays a developmental role in Or47b-expressing neurons. To rule out this possibility, we repeated the Atf3 loss-of-function experiment using Orco-GAL4. Orco-GAL4 begins to drive expression in antennal OSNs at roughly 80 hours APF, after olfactory development is complete^24^. Like the result with Peb-GAL4, knockdown of Atf3 using Orco-GAL4 eliminates Or47b expression, and this loss of Or47b expression can be partially rescued by re-introduction of Atf3 (Fig. 3D).

In further confirmation of Atf3′s role in OR expression, we performed an RNA *in situ* hybridization experiment using mixed riboprobes against both Or92a and Or47b on Atf3-null mutants. Although the *atf3*^76^ allele is a recessive lethal mutation^22^, we were able to obtain a few hemizygous male escapers. While Or92a expression in these mutant antennae remains intact, the expression of Or47b is completely lost (Fig. 3E, left). The combination of the *atf3*^76^ allele with the Atf3::eGFP BAC transgene, however, rescues both the lethality and the expression of Or47b (Fig. 3E, right).

Finally, because we observed a population of OSNs labeled by Atf3::eGFP and not by Or47b-G4>TdTomato (Fig. 3B), we asked whether Atf3 is involved in the expression of other ORs. We knocked-down the expression of Atf3 in most OSNs using Orco-GAL4 (Orco-G4>atf3-IR-1) and performed antennal RNA *in situ* hybridizations using riboprobes specific to several more ORs known to be expressed in the adult antenna (i.e., Or13a, Or22a, Or22b, Or42b, Or43b, Or59b, Or85b, Or98a, and Orco). Of these, only Or43b is lost upon knockdown of Atf3 like Or47b (Fig. 3F).

## Discussion

Here we report a simple proof-of-concept for a two-tiered, miRNA-based approach for accelerating the process of genetic screening in *Drosophila*. We generated a collection of transgenic fly stocks that permit the tissue-specific over-expression of miRNAs. Since miRNAs down-regulate their targets, we reasoned that miRNAs could be used for a pooling pre-screen that points the way to a much smaller list of RNAi stocks for follow-up screening than would normally be necessary. We demonstrated this screening method in the olfactory system where we identified a novel role for the transcription factor Atf3 in the expression of the socially relevant odorant receptor Or47b.

In addition, Eip93f, the only other transcription factor known to be involved in Or47b expression, is also a predicted target of some of the miRNAs we found to cause loss of Or47b expression. In other words, our screening strategy pointed both to a known transcription factor and, in very few genetic crosses, a previously unknown player in OR expression, Atf3.

The clear advantage to a two-tiered miRNA-based screening strategy is a reduction in the total number of crosses necessary to identify a gene associated with a specific phenotype. Genetic screening always depends on a limited resource—the patience and endurance of the researcher. In traditional genetic screens the assay for the screened phenotype must be as simple and streamlined as possible to minimize researcher fatigue. In our proof-of-principle screen in the olfactory system, we were able to identify Atf3 in roughly 160 genetic crosses. With this level of acceleration, it becomes feasible to design screens with more complicated or time-consuming assays.

Despite providing ample evidence of a role for Atf3 in Or47b expression, it remains unclear whether Atf3 is acting directly on the Or47b promoter. Miller and Carlson generated a series of GAL4 lines from truncated Or47b promoters. They observed that promoters of 7.6 kilobases down to 419 base pairs drive expression in a small population of neurons in the distolateral antenna similar to the RNA *in situ* pattern circled in blue in Fig. 1F. Further truncation expands the range of labeled cells into the proximal antenna and the maxillary palps^25^. Their results suggest the existence of an essential repressor binding site in the Or47b promoter between −419 and −342 bp from the transcription start site (TSS) and an essential activator binding site between −219 bp and −119 bp from the TSS. Unfortunately, there is no clear binding site in the Or47b promoter that matches the only published^23^ consensus sequence recognized by *Drosophila* Atf3, TGACGTCA. Thus, further experiments will be necessary to determine the exact mechanism of action by which Atf3 regulates Or47b expression.

Although our proof-of-concept screen was successful, it has not escaped our attention that miRNA-based screening is inherently biased and will not be suitable for use in every tissue or for every phenotype. One type of bias stems from our strict use of endogenous *Drosophila* miRNAs rather than designing short synthetic miRNAs that may allow targeting of a wider range of genes. Even with a collection of synthetic miRNAs, though, any form of miRNA-based screening would preferentially identify genes with longer open reading frames and longer 3′-UTRs. In fact, as is the case with traditional RNAi technology, some genes may not be accessible to miRNA-based knockdown at all. Another potential problem with a miRNA-based approach is the case where a single miRNA produces a synthetic phenotype that is not attributable to a single or predominant target mRNA. Still, we hope that the miRNA-based screening method we describe here will be another useful addition to the geneticist’s toolbox.

## Methods

### *Drosophila* stocks

All stocks were maintained at 25 °C on conventional media. For the two screening steps, virgin females (Peb-GAL4, UAS-Dcr2; Or47b>CD8::GFP, Or92a>CD8::GFP) or Orco-GAL4 (BDSC 26818), were mated to either UAS-miRNA or UAS-IR (inverted repeat) males. The crosses were maintained at 25 °C until being shifted to 27 °C during pupation to enhance GAL4 expression. All UAS-miRNA and UAS-IR lines that were found to reduce the expression of the GFP reporters were crossed and checked 2–3 times each to ensure reproducibility. Two different UAS-atf3-IR lines (IR-1 = BDSC 26741, IR-2 = VDRC 105036) produced identical phenotypes.

### Immunostaining

Brains were dissected and stained as previously described^26^ 3–4 days after eclosion with an anti-GFP antibody (1:1000, Molecular Probes) and the monoclonal antibody nc82 (1:50, DHSB), which recognizes neuropil. Frozen antennal sections were stained as described previously^24^ using an anti-GFP antibody (1:1000, Molecular Probes), and an anti-Orco antibody (1:5000) raised against the peptide (SSIPVEIPRLPIKS) by AbFrontier.

### *In situ* hybridization

*In situ* hybridization on frozen antennal sections was performed as previously described^27^ using probes generated by *in vitro* transcription from cDNAs cloned using standard methods into the pGEMT-Easy vector (Promega, USA).

### Quantitative PCR

RNA was isolated from approximately 200 dissected antennae per genotype using the easy-BLUE kit (Intron, South Korea) and treated with RNAase-free DNAase I (Takara, Japan). Antennal cDNAs were synthesized adding 2 *μ*g of total antennal RNAs to the SuperScript III First-strand Synthesis System (Invitrogen, USA). Quantitative PCR was performed using the SYBR Green reagent on a StepOne Plus Sequence detection system (Applied Biosystems, USA). Relative concentrations were calculated with the 2^−ΔΔ^Ct method using rp49 as a control and statistical significance was determined using a t-test. Primer sequences are included in Supplemental Table 2.

## Additional Information

**How to cite this article**: Bhat, S. and Jones, W. D. An accelerated miRNA-based screen implicates Atf-3 in *Drosophila* odorant receptor expression. *Sci. Rep.* **6**, 20109; doi: 10.1038/srep20109 (2016).

## Supplementary Material

Supplementary Table 1

Supplementary Table 2
